# Author Correction: FANCD2–FANCI surveys DNA and recognizes double- to single-stranded junctions

**DOI:** 10.1038/s41586-025-08763-z

**Published:** 2025-02-17

**Authors:** Pablo Alcón, Artur P. Kaczmarczyk, Korak Kumar Ray, Themistoklis Liolios, Guillaume Guilbaud, Tamara Sijacki, Yichao Shen, Stephen H. McLaughlin, Julian E. Sale, Puck Knipscheer, David S. Rueda, Lori A. Passmore

**Affiliations:** 1 MRC Laboratory of Molecular Biology, Cambridge, UK; 2 Department of Infectious Disease, Faculty of Medicine, Imperial College London, London, UK; 3 MRC Laboratory of Medical Sciences, London, UK; 4 Oncode Institute, Hubrecht Institute–KNAW and University Medical Center Utrecht, Utrecht, The Netherlands

**Keywords:** DNA damage and repair, Single-molecule biophysics, Cryoelectron microscopy, DNA, Stalled forks

Correction to: *Nature* 10.1038/s41586-024-07770-w Published online 31 July 2024

In the version of the article initially published, we omitted to include in the Methods section entitled “Electron microscopy and image processing” the method for the purification of untagged D2–I protein for cryoEM. The HTML and PDF versions of the article have now been updated.

