# Supplementary material for: FANCD2–FANCI surveys DNA and recognizes double- to single-stranded junctions
Source: Nature. 2024 Jul 31;632(8027):1165–73. doi: 10.1038/s41586-024-07770-w (PMC11358013; doi:10.1038/s41586-024-07770-w)
Supplement: Supplementary file 1 — This file contains Supplementary Figs. 1–4, Tables 1 and 2 and Data 1. [file 41586_2024_7770_MOESM1_ESM.pdf]

nature portfolio

https://doi.org/10.1038/s41586-024-07770-w

Supplementary information

FANCD2–FANCI surveys DNA and recognizes double- to single-stranded junctions

In the format provided by the
authors and unedited

Supplementary Figures

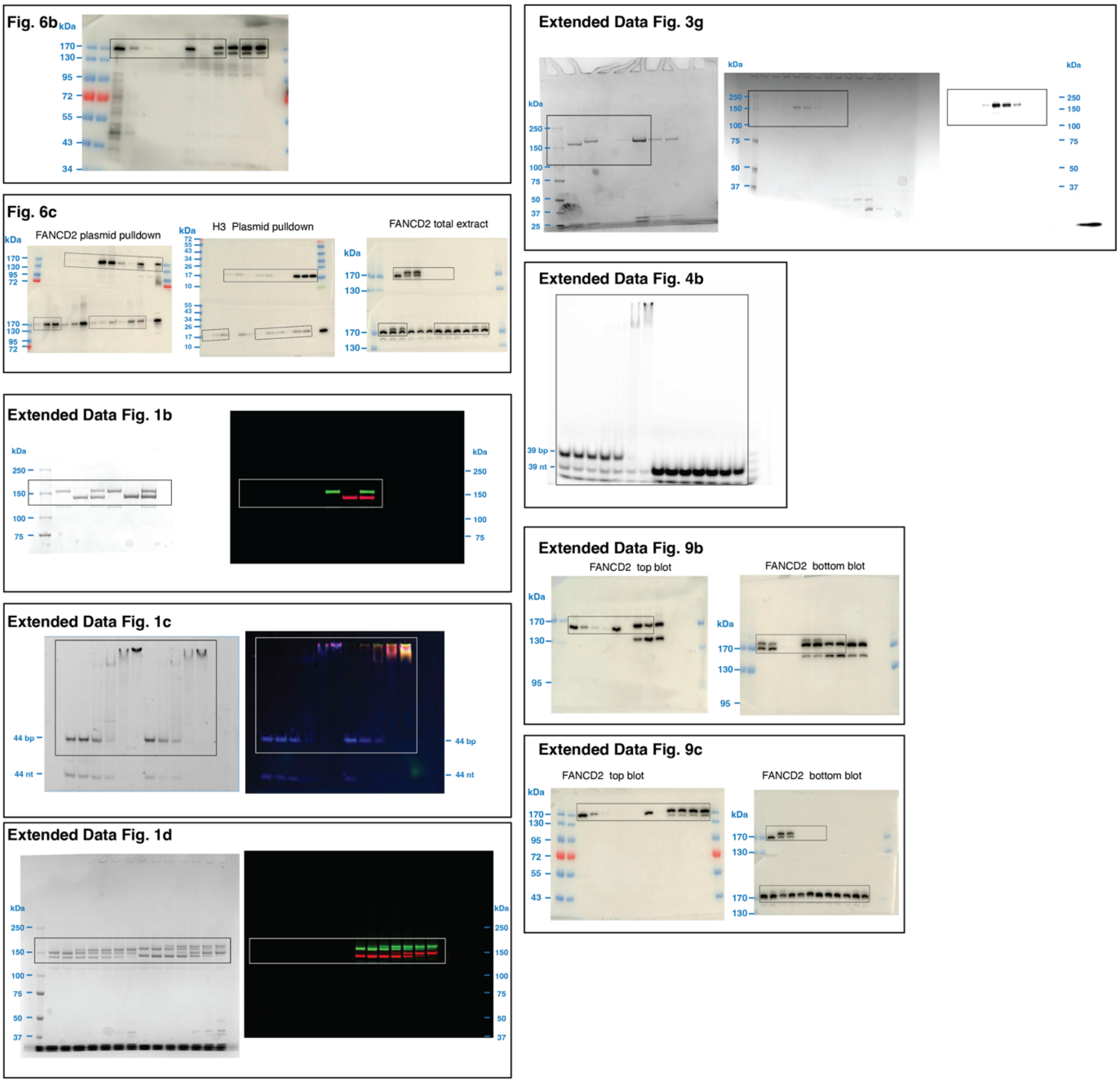

Supplementary Fig. 1. Uncropped gels

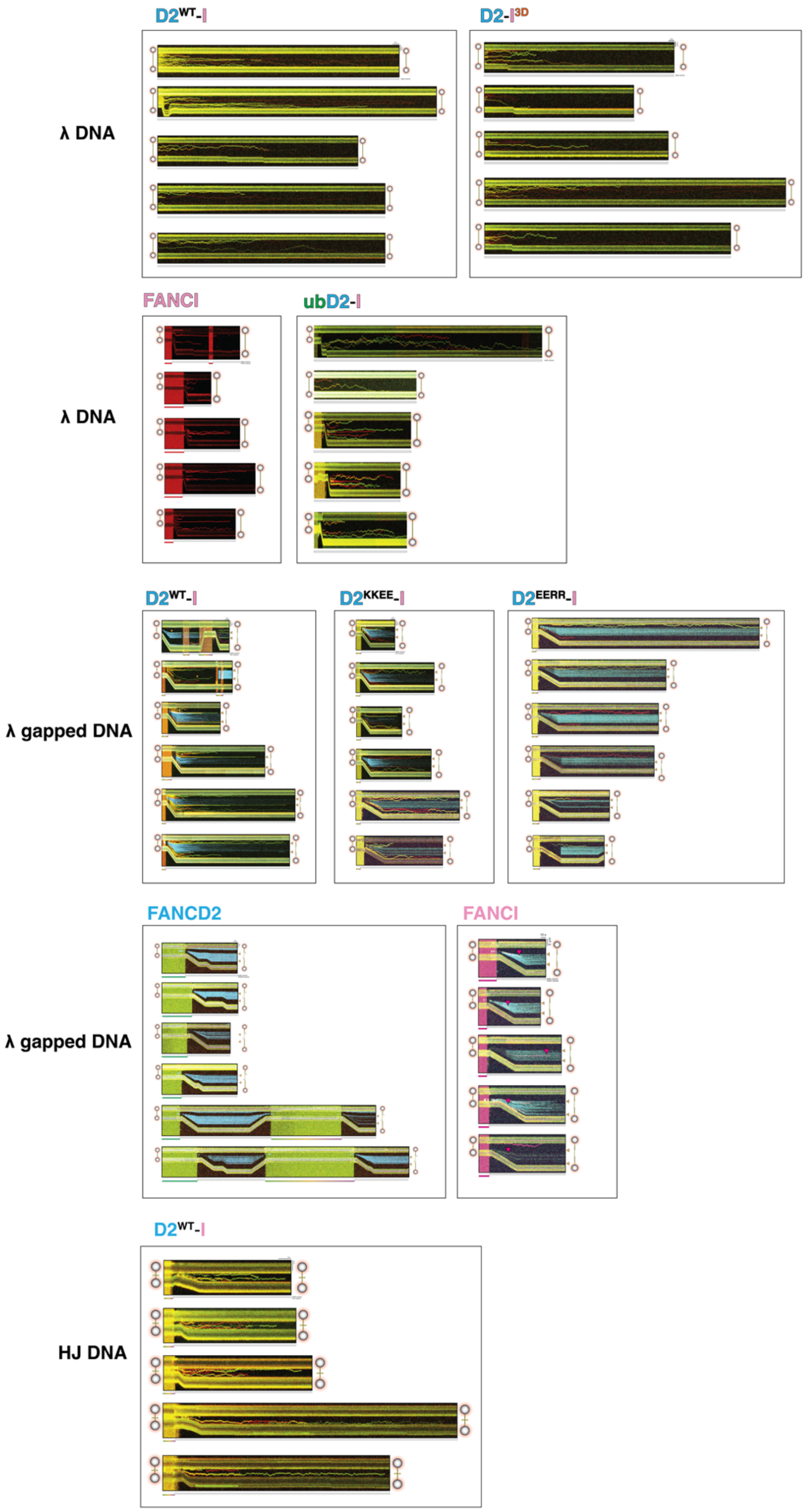

Supplementary Fig. 2. Gallery of representative kymographs.

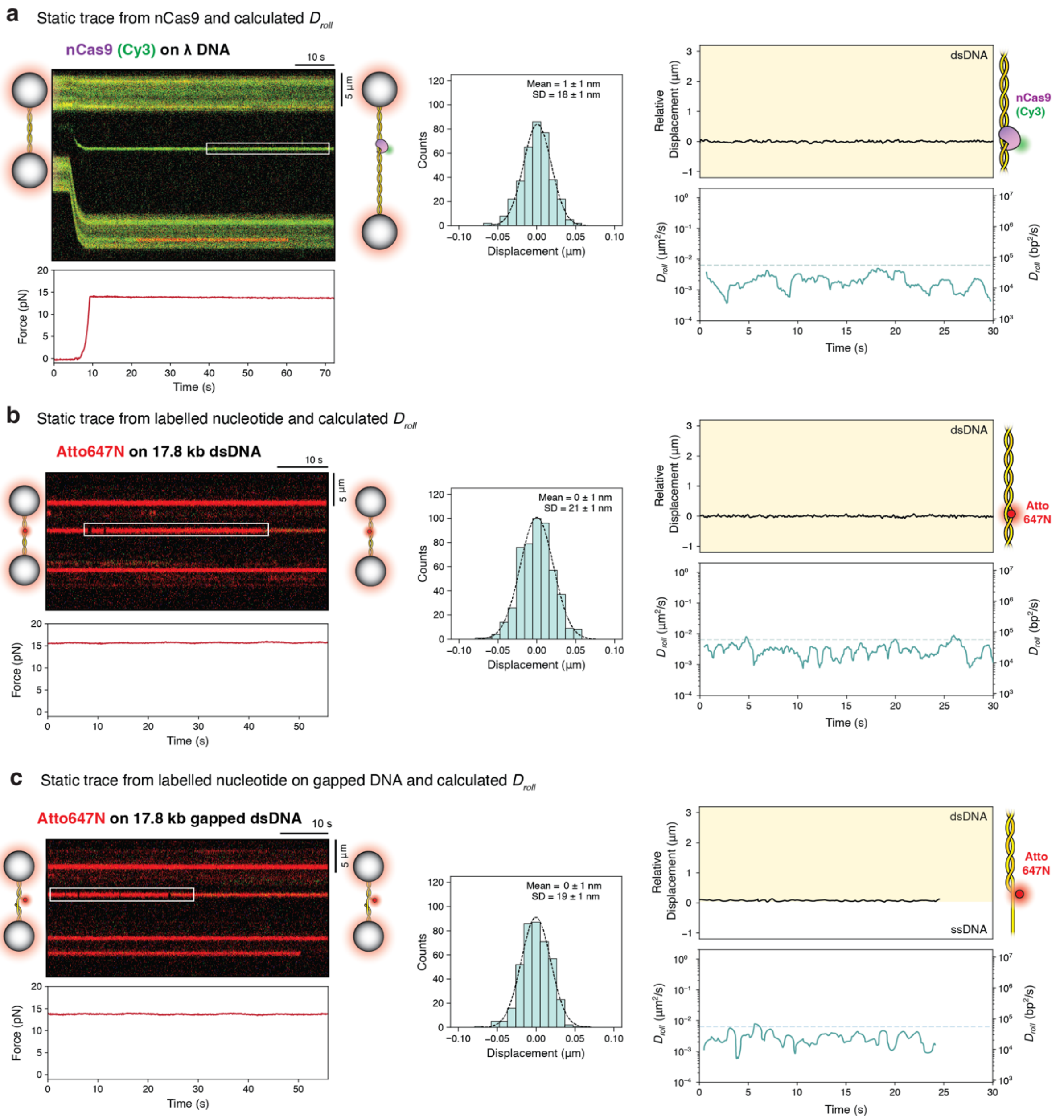

Supplementary Fig. 3. Characterization of the spatial resolution of the single-molecule assay. a-c, Utilizing a single-particle tracking algorithm, we detected the trajectories of three fiduciary static molecules on DNAs tethered to the optical traps at 15 pN force: a Cy3-labelled nCas9 on λ DNA (a), Atto-647N dye on double-stranded 17.8 kb DNA (b), and an Atto-647N dye on a 17.8 kb DNA containing a ssDNA gap (c). Dye positions were tracked over time (white rectangle). (Middle panels) Histograms of displacements over single time points for the tracks were fit to Gaussian distributions. As expected, the means of these distributions were nearly zero, showing that there was no systematic bias in our tracking assays. The widths, i.e., the standard deviations of the distributions, were ~20 nm, reflecting the precision in our detection of consecutive static positions. Thus, we could estimate that the spatial resolution of our assays is ~20 nm on average (a-c, central panels). This analysis showed that bead diffusion in the trap can lead to apparent displacements over short timescales, even for static molecules. Thus, to distinguish D2-I stalling from small displacements caused by frustrated diffusion of D2-I around obstacles, we employed a

rolling diffusion analysis approach (right panels). Briefly, we computed mean squared displacements (MSDs) over short windows (16-time points) rather than over the entire trajectory, yielding the rolling diffusion coefficients (D_{roll}) for these specific windows. Employing rolling windows over the entire trajectory, we could estimate how the diffusion of D2-I changed over the course of the trajectory. For our fiduciary static molecules, we observed that D_{roll} is consistently low ($<6.4\times10^{-3}$ $\mu m^2/s$). We therefore used this value as a threshold (D_{th}) below which we could consider D2-I to be static in our assays.

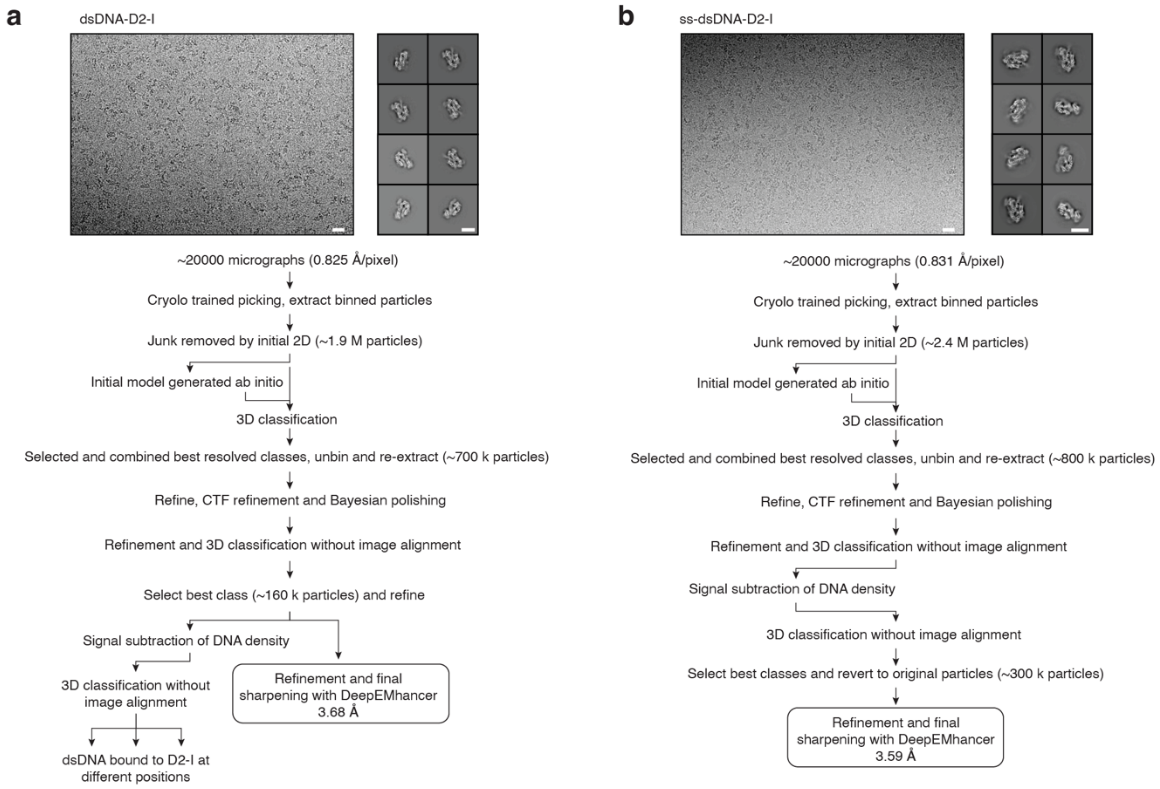

Supplementary Fig. 4. CryoEM data processing pipelines. Representative micrographs, 2D class averages and flowcharts outlining the data processing of D2-I bound to dsDNA (**a**) and ss-dsDNA (**b**). All image processing was performed in RELION unless otherwise stated. Scale bars on micrographs, 20 nm. Scale bars on 2D class averages, 10 nm.

Supplementary Table 1. Primers and oligonucleotides used in this study.

Name	Sequence (5′ to 3′)	nt
P1 (3′ FAM)	ATCGATGTCTCTAGACAGCTGCTCAGGATTGATCTGTAATGGCC/36-FAM/	44
P7	GGCCATTACAGATCAATCCTGAGCAGCTGTCTAGAGACATCGAT	44
J1	TTTTTTTTTTTTTTTTTTTTTTTTTTTTTTATCGATGTCTCTAGACAGCTGCTCAGGATTGATCTGTAATTTTTTTTTTT TTTTTTTTTTTTTTTTTTT	99
J2	ATCCTGAGCAGCTGTCTAGAGACATCGAT	29
P1-29	ATCGATGTCTCTAGACAGCTGCTCAGGAT	29
biotin end cap 1	AGGTCGCCGCCCGGAGTTGAACGT^{biot}T^{biot}TT^{biot}TT^{biot}ACGTTCAACTCC	41
biotin end cap 2	GGGCGGCGACCTCAAGTTGGACAAT^{biot}TT^{biot}TT^{biot}T^{biot}TGTCCAACTTG	41
crRNA 1	CAGATATAGCCTGGTGGTTCGUUUUAGGAGCUAUGCUGUUUUG	43
crRNA 2	GGCAAUGCCGAUGGCGAUAGGUUUUAGGAGCUAUGCUGUUUUG	43
tracrRNA	GGACAGCAUAGCAAGUUAAAAUAAGGCUAGUCCGUUAUCAACUUGAAAAAGUGGCACCGAGUCGGUGCUUUUU	73
HJ handle primer 1	(5′-Biot)T^{Biot}CAT^{Biot}CT^{Biot}GAAACAGCAGCGGA	20
HJ handle primer 2	CTTACAGATG(ab)CGCACGAAAAGCATCAGGTC	31
HJ oligo 1	ACATCTGTAAGAGTCTGCAGTTGAGTCCTTGCTAGGACGGACGAAGTCCGTCCTAGCAAGGGGCTGCTACCGGAAG	76
HJ oligo 2	ACATCTGTAAGCTTCCGGTAGCAGCCTGAGCGGTGGATGAACGAAGTTCATCCACCGCTCAACTCAACTGCAGACT	76
ggKKAA gBlock	CTGATCCACTCCACTAACTCCAAGAACCGCAAACAGACCGAGAAAGTTCTGGCGTCTAAGATTGCGCTGGGCTGTATGCC GGAACAACTGATGCAGAATGCGTTCCAGAACCACTCTATGGTGATC	126
ggAARR gBlock	CTGATCCACTCCACTAACTCCAAGAACCGCGCGCAGACCGAGGCGGTTCTGCGTTCTAAGATTCGTCTGGGCTGTATGCC GGAACAACTGATGCAGAATGCGTTCCAGAACCACTCTATGGTGATC	126
ggAAAA gBlock	CTGATCCACTCCACTAACTCCAAGAACCGCGCGCAGACCGAGGCGGTTCTGGCGTCTAAAATTGCGCTGGGCTGTATGCC GAACAACTGATGCAGAATGCGTTCCAGAACCACTCTATGGTGATC	126
ggKKEE gBlock	CTGATCCACTCCACTAACTCCAAGAACCGCAAACAGACCGAGAAAGTTCTGGAGTCTAAGATTGAGCTGGGCTGTATGCC GAACAACTGATGCAGAATGCGTTCCAGAACCACTCTATGGTGATC	126
ggEERR gBlock	CTGATCCACTCCACTAACTCCAAGAACCGCGAGCAGACCGAGGAGGTTCTGCGTTCTGCGATTCGTCTGGGCTGTATGCC GAACAACTGATGCAGAATGCGTTCCAGAACCACTCTATGGTGATC	126
ggEEEE gBlock	CTGATCCACTCCACTAACTCCAAGAACCGCGAGCAGACCGAGGAGGTTCTGGAGTCTAAAATTGAGCTGGGCTGTATGCC GAACAACTGATGCAGAATGCGTTCCAGAACCACTCTATGGTGATC	126
xlKREE gBlock	ATCCTGTACACTACCCAGACCAACTCTAGCAAGAAACAGGCTGAACGTGTACTGGAGAACAAGATCGAGTCTGGCTTCAT CTGGATCAACTGCTGCAGAATGCCTTCCGTAACCATTCCCAAGTTCTG	129
xlEERR gBlock	ATCCTGTACACTACCCAGACCAACTCTAGCAAGGAGCAGGCTGAAGAGGTACTGCGTAACAAGATCCGTTCTGGCTTCAT CTGGATCAACTGCTGCAGAATGCCTTCCGTAACCATTCCCAAGTTCTG	129
xlEEEE gBlock	ATCCTGTACACTACCCAGACCAACTCTAGCAAGGAGCAGGCTGAAGAGGTACTGGAGAACAAGATCGAGTCTGGCTTCAT CTGGATCAACTGCTGCAGAATGCCTTCCGTAACCATTCCCAAGTTCTG	129
K400A-K404A mutant (Watson)	GAATCTTGCTCCTCAATACCGCCTCAGTTTGCGCCCTGTTCTTGCTGTTTGTAG	54
K400A-K404A mutant (Crick)	GAATCTTGCTCCTCAATACCGCCTCAGTTTGCGCCCTGTTCTTGCTGTTTGTAG	54
R407A-R411A mutant (Watson)	CATGCAGCCCAGGGCAATCTTGCTCGCCAATACCTTCTCAGTTTGC	46
R407A-R411A mutant (Crick)	GCAAACTGAGAAGGTATTGGCGAGCAAGATTGCCCTGGGCTGCATG	46
K400E-K404E mutant (Watson)	CTACAAACAGCAAGAACAGGGAGCAAACTGAGGAGGTATTGAGGAGCAAGATTC	54
K400E-K404E mutant (Crick)	GAATCTTGCTCCTCAATACCTCCTCAGTTTGCTCCCTGTTCTTGCTGTTTGTAG	54
R407E-R411E Mutant (Watson)	CTGGCATGCAGCCCAGCTCAATCTTGCTCTCCAATACCTTCTCAGTTTGCTTCCT	55
R407E-R411E Mutant (Crick)	AGGAAGCAAACTGAGAAGGTATTGGAGAGCAAGATTGAGCTGGGCTGCATGCCAG	55
K400A-K404A-R407A-R411A (W)	GGCATGCAGCCCAGGGCAATCTTGCTCGCCAATACCGCCTCAGTTTGCGCCCTGTTCTTGCTGTT	65
K400A-K404A-R407A-R411A (C)	AACAGCAAGAACAGGGCGCAAACTGAGGCGGTATTGGCGAGCAAGATTGCCCTGGGCTGCATGCC	65
K400E_K404E_R40E_R411E (W)	CTGGCATGCAGCCCAGCTCAATCTTGCTCTCCAATACCTCCTCAGTTTGCTCCCTG	56
K400E_K404E_R40E_R411E (C)	CAGGGAGCAAACTGAGGAGGTATTGGAGAGCAAGATTGAGCTGGGCTGCATGCCAG	56
K563 (Watson)	GAGCTGGAGAGCCACCTCCTGATCACCATGT	31

K563 (Crick)	ACATGGTGATCAGGAGGTGGCTCTCCAGCTC	31
cNLA	ATCAGTACTTGTCAACACGAGCAGCCCGTATATTCTCCTACAGCACTA	48
cNLB	ATCAGCGTTCGATGCTTCCGACTAATCAGCCATATCAGCTTACGACTA	48
30-48-cNLB	ATCGATGTCTCTAGACAGCTGCTCAGGATTATCAGCGTTCGATGCTTCCGACTAATCAGCCATATCAGCTTACGACTA	78
38-cNLB	GATGCTTCCGACTAATCAGCCATATCAGCTTACGACTA	38
38P-cNLB	^{phospho}GATGCTTCCGACTAATCAGCCATATCAGCTTACGACTA	38

5'-Biotin phosphoramidite (**5'-Biot**) (Glen Research, 10-5950-95)
Biotin-dT phosphoramidite (**T^{Biot}**) (Glen Research, 10-1038-95). (primers home made by A-C Declais, Tbiot in end-caps is within the oligos ordered by IDT)
dSpacer CE phosphoramidite (**ab**) (Glen Research, 10-1914-90)

Supplementary Table 2. Analysis for cell survival data.

R package CFAssay
Two-way analysis for cell survival data
Fits LQ model with maximum likelihood

	WT	Control	FANCD2	K400A_K404A	R407A_R407A	K400E_K404E	R407E_R411E	K400A_K404A_R407A_R407A	K400E_K404E_R407E_R407E	K563R
WT	-	1.80E-01	1.17E-05	1.34E-07	3.21E-01	5.84E-03	8.08E-01	1.57E-09	3.73E-07	2.21E-16
ChFANCD2 in D2-/- (Control)	-	-	5.29E-05	1.68E-08	7.93E-01	1.33E-04	5.82E-01	1.44E-09	1.03E-07	1.42E-23
FANCD2	-	-	-	7.91E-01	1.55E-09	1.86E-02	2.03E-09	6.34E-02	2.56E-01	1.25E-03
K400A_K404A	-	-	-	-	9.43E-11	3.73E-04	3.34E-13	4.20E-02	5.44E-01	5.56E-05
R407A-R411A	-	-	-	-	-	1.18E-04	5.19E-01	2.71E-13	2.47E-10	4.79E-10
K400E_K404E	-	-	-	-	-	-	1.27E-05	3.76E-07	9.24E-04	2.25E-09
R407E-R411E	-	-	-	-	-	-	-	1.94E-15	1.07E-12	5.14E-13
K400A_K404A_R407A_R407A	-	-	-	-	-	-	-	-	5.87E-02	1.49E-01
K400E_K404E_R407E_R407E	-	-	-	-	-	-	-	-	-	7.85E-04
K563R	-	-	-	-	-	-	-	-	-	-

Supplementary Data 1. Protein sequences. Monoubiquitination sites are marked in green and phosphorylation sites (mutated to D in the FANCI-3D version) are marked in orange. The KR helix in FANCD2 is shown in bold with K/R residues mutated in assays marked in magenta.

ggFANCD2_SII_ybbR

MVSKRKLSKIDAAEESSKTDLQSRCPETKRSRISDKRAPSQGGLENEGVFEELLRTSGIILKVGEGQNEIAVDQTAFQKKL
RVALEKHPSYPGVVNEFISGLESHIKDRSQFKNCLLPCTPARTEGSRTLVHSYCESLIKLLLGIKILQPAVVTLLLEKIPE
FFFDVVGTFGTNFPRLIVNQFKWLDGLLDSQDLVKKLMQMLSVSPVPIQHDIITSLPEILEDSQQNEVARELSCLLKQGRR
LTVPILDALSRLDLDAELLAKVRQSAMTIVPSVKLEDLPVVIKFILHNVKAADAVEVISDLRKSLDLSSCVLPLQLLGSQR
KLKSQAQASSSMSQVTTSQNCVKLLFDVIKLAVRFQKDVSEAWIKAIENSTSVSDHKVLDLIVLLLIHSTNS**KNRKQTEKV**
LRSKIRLGCMPEQLMQNAFQNHSMVIKDFFPSILSLAQTFLHSAHPAVVSFGSCMYKQAFAVFDSYCQQEVVCALVTHVCS
GNETELDISLDVLTDLVILHPSLLLRYATFVKTILDSMQKLNPCQIRKLFYILSTLAFSQRQEGSYIQDDMHMVIR**K**WLSS
SVPNHKQMGIIGAVTMMGSVALKRNEADGGLLERPELSIECDGQLSTLLDLVGFCCEQTPEVLALYYDELANLIEKQKGNL
DLQLLDKFGKSLVEDFPNDFVVDLSPTVDGSFLFPVKSLYNLDEDETQGAIAINLLPLVSQSEPGRVADEMSNSRKRVVSP
ICLSPCFRLLRLYTGEQNNGSLEEIDALLGCPLYLTDLEVEGKLDSLSKQEREFLCSLLFYALNWFREVVNAFCQQQDAEM
KGKVLTRLQNITELQNVLGKCLAATPGYVPPPATFDSEAPEGVPSINAGGPVRKKNGKKRKSDSSKACSAERTQADESSDG
NQPDTELSELEKSAAEKETGNPLAQLQSYRPYFRELDLEVFSVLHCGLLTKSILDTEMHTEASEVVQLGPAELCFLLDDMC
WKLEHVLTPGSTRRVPFLKERGNKDVGFSHLCQRSPKEVAVCVVKLLKPLCNHMENMHNYFQTVIPNQGVVDESGLNIQEY
QLMSSCYHQLLLAFRLLFAWSGFSQHENSNLLRSALQVLADRLKPGETEFLPLEELISESFQYLLNFQASIPSFQCAFILT
QVLMAISEKPMTGWKREKMASLAKQFLCQSWMKPGGDREKGSHFNSALHTLLCVYLEHTDNILKAIEEISSVGVPELINSA
KDGCSSTYPTLSRQTFPVFFRVMMAQLESSVKSIPAGKPSDSGEVQLEKLLKWNIAVRNFHILINLVKVFDSRPVLSICLK
YGRLFVEAFLKLAMPLLDHSFKKHRDDVQSLLKTLQLSTRQLHHMCGHSKIHQDLGLTNHVPLLKKSLEQFVYRVKAMLAF
NHCQEAFWVGVLKNRDLQGEEILSQASAAPEEDSAEGSEEDTEDSAAEEPDGTDSDSGGAGRLEVLFQGPWSHPQFEKGSA
GSAAGSGAGWSHPQFEKDSLEFIASKLA*

ggFANCI_His_ybbR

MAQRILQLAAEGSPERLQEALQGLTEGELGDMVTRQALRGRETAALLKGIFKGSPCSQQSGVLRRLQVYKHCVSLVESGDL
HVGKVSEIIGLLMLEARQLPGHALAELATLFVEVIKRGSLSNGKSLELFSTVLTALSNSKESLAYGKGELNGEEFKKQLIN
TLCSSKWDPQCVIHLANMFRDIPLSGEELQFVVEKVLRMFSKLDLQEIPPLVYQLLLLSAKGSKKTVLEGIISFFNQLDKR
QKEEQRVPQSADLEVATVPLDQLRHVEGTVILHIVSAINLDQDIGEELIKHLKTEQQKDPGKALCPFSVSLLLSTAVKHRL
QEQIFDFLKTSITRSCKDLQILQASKFLQDLCPQQYDVTAVILEVVKNSAFGWDHVTQGLVDLGFSLMESYEPKKSFGGKA
AETNLGLSKMPAQQACKLGASILLETFKVHEPIRSDILEQVLNRVLTKAASPVSHFIDLLSNIVVSAPLVLQNSSSRVTET
FDNLSFLPIDTVQGLLRAVQPLLKVSMSVRDSLILVLQ**K**AIFSRQLDARKAAVAGFLLLLRNFKILGSLTS**S**QC**S**QAIGA**T**
QVQADVHACYNSAANEAFCLEILGSLRRCLSQQADVRLMLYEGFYDVLRRNSQLASSIMETLLSQIKQYYLPQQDLLPPLK
LEGCIMAQGDQIFLQEPLAHLLCCIQHCLAWYKSTVHLCKGAEDEEEEEDVGFEQNFEEMLESVTRRMIKSELEDFELDKS
ADFSPSSGVGVKNNIYAIQVMGICEVLIEYNFKIGNFSKNKFEDVLGLFTCYNKLSEILKEKAGKNKSTLGNRIARSFLSM
GFVSTLLTALFRDNAQSHEESLAVLRSSTEFMRYAVSVALQKVQQLEEMGQTDGPDGQNPEKMFQNLCKITRVLLWRYTSI
PTAVEESGKKKGKSISLLCLEGLLRIFNTMQQLYAARIPQFLQALDITDGDAEEADINVTEKAAFQIRQFQRSLVNQLSSA
EDDFNSKETQLLITILSTLSKLLDPGSQQFLQFLTWTVKICKENALEDLSCCKGLLTLLFSLHVLYKSPVSLLRELAQDIH
ACLGDIDQDVEIESRSHFAIVNVKTAAPTVCLLVLGQADKVLEEVDWLIKRLTILGSDTSEDSTQASNQTQALEKGVILQL
GTLLTVFHELVQTALPAGSCVDSLLRSLSKTYAILTSLIKHYIQACRSTSNTVPGRLEKLVKLSGSHLTPQCYSFITYVQN
IHSESLSFAEEKKKKKKEDETAVVSTVMAKVLRDTKPIPNLIFAIEQYEKFLIHLSKKSKVNLMQYMKLSTSRDFRINASM
LDSVLQEQNTEDAENEPDNNQSGTAEQPDENQEPQKKRRRKKGSGSHHHHHHDSLEFIASKLA

xlFANCD2_SII

MVAKRKLSRSDDREESFTADTSKNKKCRTSSKKSKALPQDGVVENDSIFVQLLKSSGMTLKCGDRQNEIAVDQAVFQRKLQ
QALRKHPRHPNVIQEFISGLESHIEDRDIFRNCLLPCGNRQETEASTMTGSFHDSLIKLLLGTDILQESVINTLFEKLPEF
LYDSVGSDGISIPRLIINQFKWLDRILDCKDLTLKIMQLISVAPVDIQHDIVTSLPEILEDSQHNDVARELNSLLQQNTQL
TVPILDALSSLNINADLLSEVRQSVMSTLSAVELEDLPVIIKFILHAVTPSDALEVISELRKKLDLESCSSLEQIYATQSK
ERNKPHAGSSVNKTKSSDCVSLMMDVIKSSVRFQKHTSEAWMKAIENVDTVGDHKVSDLIVLLILYTTQTNSSK**K**QAE**R**VL
RNKI**R**SGFILDQLLQNAFRNHSQVLRDYFPSILSLAQSMLRSAEQSVVSFGSLMYKSAFSSFDSYCQQEVVGALVTHVCSG
YAAEVDVSLDVLTDLVSSHAAAVALYAVFVKGILDYLDNLNAQQIRKLFHILSVLAFSRGQEGGHIQDDMFIVIRKQLSST
VLKYKRIGIIGAVQMVGSMAMNKKHGSKHPENKPLSAETFRQVTALLELLQTCSERVAEASALYYDELSSLVQKRNLDPQV
MSLVGKTVLTDFQDDFVEDLTPTEEGNYIFPLKAMYNLDEDDSQGGIAINLLPLLSQDMRNRGAEQVANKEGRVVSPICLS
PFFRLLRLCIEDQHEGNLEEIDALLGCPLYLTDLEITEKMESLSKQEREFLCSLLFFALNWFREIVNAFCKQQDPDMKGKV
LTRLQNITWLQSVLEKCLAASPGYIPSSAHFDSEPQEVLPSAIAPAPAKKAKKGKTPKSAGSKNASADSSQLEEHMDTEKS
ELEKTQPEKEKEESKPSINLNNYRAYFRELDLEVFTVLQCGLLTRSLLDSEMHTKATEVVQLGPAELVFLLEDLFRKTENI
LTSAAKRVTFLKVKKGRNLGFSQLLQKTPQEVAQAVIHLLNSLCNHIENMHNYFQTLMLENHGVVDAPGVDIKEHQYMSTC
YQLLLQVFHTLFCWNGFSQHENRKLLKSALIALAGRLKETETEPALDDLVRQSFNYLKNLHSSVPTCSSALCLTQLLIVIA
EKTNVLQYREQIASMAKQFLCQAWIQPSGEREKGIRYHENLQSLLCIYLENTDDVLKAVEDIAGVGVPELVNAAKDAASSS
YPTLTRQTFVVFFRVMMDKLEKCVKSIPNSKKAETLQEQTEQLLSWNLAVRDFHILVNLVKVFDSRPVLSICLKYGRLFVE

TFLKLGMPLLDCCFKKQREDVQSLLKTLQLSTRQLHHMCGHSKINQDTALTNHVPLLKKTLELFVYRVKAMLVLNNCQEAF
WLGNLKNRDLQGEEIVSQVSQESEAEEEQESQLPPEEEEAEEEEENEGSDKEIEGGDEDNEDEDSDLEVLFQGPWSHPQFE
KGSAGSAAGSGAGWSHPQFEK

xlFANCI_His
SHHRARIPRVNMDQKILSLAAEEQNDGLQSYLQNLKETELSEIITKHAVKGKDCGALLRGVFKGSPCSHDVAVRRRLAVYR
HCIQLVESGDLQREVASEIMGLLMLEVHHFPGASLIELANLFADAVKAGTLSNGKSLDLFPTVLTALSSKESLAYGKGELT
GEEFKKQLINSLCSSRWDPQSVIFLTSMFRDVSLSAEELQFVVEKVLRMLSKLDLQEMPPLVYQLLLLSAKGSKKNIVEGI
ITIFNDLDQKQLVQQENSESLDLEDATIPQDQLHHVEGTIILHIVFAIKLDQELGRELLKYLKAGQQGDSSKILCPFSAAL
LLSVSRIHRFQEQVFDFLKSTILRDYKDLQFHQSSKFLQDLVPQPVCVSSILLETVKNSVYGWDHVTQGLVELGFILMDSF
GPKAAFGNKVVELISVHSRTPSQQACQLGSRILLETFKVHEPIRSEILEQVLNRVITKAATPVTHFIDLLSDIVVSAPLIL
QNSSSKVTEAFDHLSFLPLTTVQGLLKAVQPLLKISMSMRDSLILVLRKAMFSSQIDARKSAVAGFLLLLRNFKVLGSLSS
SQCSQAIGASQIQVDVHMRYNAAANEAFCLEILGSLRRCLSQQADVRLMLYEGFYDVLRRNSQLASSVMQTLLSQLKRYYE
PEPDLLPPLKLEGCITAQGDHIFLQEPLAHLLCCIHHCLQWYKSSLQQHRNPDEDDDDDDQTGCQQDLNDIMESITRRMIK
CDLEDFELDKSADFSLASGVGVKNNIYAVLVMGICEVLIEYNFIIANFSKSKFEDILGLFKCCSKLSDILKEKAAKGRQPG
NNKTARSLISMTFVSTLLTALFRDSTRSHEESLSILRANVDFMRYSVCVALQKIQQLEETGVTDGPDGQNSEKMFRSLCEI
TRVLMWRYTSIPAAAENPGKKDKGKTISLLCLEGLLRVFNTVQQRYPSKIPQFLTALDALGDEDEEGSREINVTEKAAFQI
KQFQRSLINQLSGGEDDFNSKEALLLVSILSTLSRLLAPSSQQFVQMLSWTVKICKETNIEDVQFCKGIMNLLFSLHVQFK
SPVSVLRELCQDIHGHLGDIDQDIEVEKQSHFATVSLKTAAPTVTLLVLGQAAKVLEEVDWLIIKLKGLLGSEKLSTEDLT
QTSNARVPIEKAIILQLGTLLTACHELVQTALPAGSCTDTLLKELAKMYTILTSVVKYYLQICSSHGGQISARLEKLVKLS
GSHLTPQCYAFITYVQNIHSESLSLAAEKKKKKKEEALTVTSAKILRDTKPIPNLIFAIEQYEKFLIHLSKKSKVNLMQYM
KLSTSRDFRINAATLDAALQEKGSEDEENEPDNEQAVTEEESQEPKKKRRRKGSGSHHHHHH
