# Supplementary material for: FANCD2–FANCI surveys DNA and recognizes double- to single-stranded junctions
Source: Nature. 2024 Jul 31;632(8027):1165–73. doi: 10.1038/s41586-024-07770-w (PMC11358013; doi:10.1038/s41586-024-07770-w)
Supplement: Supplementary file 2 — Reporting Summary [file 41586_2024_7770_MOESM2_ESM.pdf]

nature portfolio

Corresponding author(s): Lori A Passmore and David S Rueda

Last updated by author(s): Jun 7, 2024

Reporting Summary

Nature Portfolio wishes to improve the reproducibility of the work that we publish. This form provides structure for consistency and transparency in reporting. For further information on Nature Portfolio policies, see our Editorial Policies and the Editorial Policy Checklist.

Statistics

For all statistical analyses, confirm that the following items are present in the figure legend, table legend, main text, or Methods section.

n/a	Confirmed	
☐	☒	The exact sample size (*n*) for each experimental group/condition, given as a discrete number and unit of measurement
☐	☒	A statement on whether measurements were taken from distinct samples or whether the same sample was measured repeatedly
☐	☒	The statistical test(s) used AND whether they are one- or two-sided<br>*Only common tests should be described solely by name; describe more complex techniques in the Methods section.*
☒	☐	A description of all covariates tested
☒	☐	A description of any assumptions or corrections, such as tests of normality and adjustment for multiple comparisons
☐	☒	A full description of the statistical parameters including central tendency (e.g. means) or other basic estimates (e.g. regression coefficient) AND variation (e.g. standard deviation) or associated estimates of uncertainty (e.g. confidence intervals)
☐	☒	For null hypothesis testing, the test statistic (e.g. *F*, *t*, *r*) with confidence intervals, effect sizes, degrees of freedom and *P* value noted<br>*Give P values as exact values whenever suitable.*
☒	☐	For Bayesian analysis, information on the choice of priors and Markov chain Monte Carlo settings
☒	☐	For hierarchical and complex designs, identification of the appropriate level for tests and full reporting of outcomes
☒	☐	Estimates of effect sizes (e.g. Cohen's *d*, Pearson's *r*), indicating how they were calculated

Our web collection on statistics for biologists contains articles on many of the points above.

Software and code

Policy information about availability of computer code

Data collection	EPU version 3.4.0 (Thermo Fisher Scientific) was used for all cryo electron microscopy data collection. A Typhoon Imaging System (GE Healthcare) or Gel Doc XR+ system (Bio-Rad) was used for gel imaging. Single-molecule data was collected using the commercial software Bluelake v. 1.6 (LUMICKS) that is fully compatible with the instrument (correlative optical tweezer and confocal microscope C-TRAP). Measurements of individual DNA molecules were saved as .H5 Hierarchical Data Format. All H5-files contain force and position data of optically trapped beads, as well as pixel values of the recorded 3-color confocal images. The exported files contain additional meta-data such as: experimental description, status of the microfluidic system, laser powers, laser coordinates, camera settings.
Data analysis	Relion v4.0, MotionCor2, CTFFIND4, crYOLO 1.7.6, 3DFSC, Coot, Phenix 1.20.1, UCSF Chimera 1.15, ChimeraX-1.16.1, ISOLDE, DeepEMhancer, Prism 10, switchANALYSIS 1.9.0.33 were used for data analysis.<br>Single-molecule data were processed and analyzed using custom-made scripts written in Python v. 3.9 using Pylake 1.2.1/Numpy 1.26.0/ Matplotlib 3.7.2/Scipy 1.11.3/Peakutils 1.3.4. packages<br>Each H5-file was processed in a separate Jupyter Notebook - an interactive platform to visualize the results of the Python script. Within a single Jupyter Notebook, the fluorescent data was rendered into an RGB image, was correlated with the force measurement, subsequently underwent the single-particle tracking and mean-square-displacement (MSD) analysis. A representative Jupyter Notebook that includes all the above features is available at www.github.com/singlemoleculegroup.<br>Selected images rendered in Jupyter Notebook into .TIFF images were cropped in ImageJ 2.1<br>Commercial software Wavemetrics IGOR 8 was used to generate final plots (force-distance curves, 1D trajectories, MSD plots and histograms). To do so, the output of each Jupyter Notebook (in the .CSV format) was imported to IGOR's workspace.

For manuscripts utilizing custom algorithms or software that are central to the research but not yet described in published literature, software must be made available to editors and reviewers. We strongly encourage code deposition in a community repository (e.g. GitHub). See the Nature Portfolio guidelines for submitting code & software for further information.

Data

Policy information about availability of data

All manuscripts must include a data availability statement. This statement should provide the following information, where applicable:

- Accession codes, unique identifiers, or web links for publicly available datasets
- A description of any restrictions on data availability
- For clinical datasets or third party data, please ensure that the statement adheres to our policy

The model of FANCD2 in complex with FANCI (PDB 6TNG) was used as initial reference for model building into cryoEM maps. The models and maps generated in this study have been deposited to PDB and EMDB and assigned the following accession codes: PDB ID 9FFF, EMD-50355 for dsDNA-D2-I; PDB ID 9FFB, EMD-50353 for ss-dsDNA-D2-I. All original gels shown in this study are provided as Source Data. Other raw data is accessible on Zenodo at DOI: 10.5281/zenodo.11521474. Code for analysis of single molecule data are freely available (https://github.com/singlemoleculegroup). Correspondence and requests for materials should be addressed to L.A.P. or D.S.R.

Research involving human participants, their data, or biological material

Policy information about studies with human participants or human data. See also policy information about sex, gender (identity/presentation), and sexual orientation and race, ethnicity and racism.

Reporting on sex and gender	Not applicable
Reporting on race, ethnicity, or other socially relevant groupings	Not applicable
Population characteristics	Not applicable
Recruitment	Not applicable
Ethics oversight	Not applicable

Note that full information on the approval of the study protocol must also be provided in the manuscript.

Field-specific reporting

Please select the one below that is the best fit for your research. If you are not sure, read the appropriate sections before making your selection.

☒ Life sciences ☐ Behavioural & social sciences ☐ Ecological, evolutionary & environmental sciences

For a reference copy of the document with all sections, see nature.com/documents/nr-reporting-summary-flat.pdf

Life sciences study design

All studies must disclose on these points even when the disclosure is negative.

Sample size	Sample sizes were selected based on previous experience and published studies to evaluate reproducibility of assays. For quantified results, the number of replicates is indicated in the Figure legends. The number of micrographs in our cryoEM data collection was chosen accordingly to obtain the required resolution.
Data exclusions	1) Kymographs that contained too many diffusing species were excluded from quantifications because the protein molecules were colliding with each other, obscuring the results of a single-particle tracking algorithm.<br>2) Trajectories of protein diffusing on the DNA were not included in the statistics if the diffusion was shorter than 5 seconds (not enough datapoints to fit linear mean-square-displacement).<br>3) For the colony survival assay (DT40), some conditions were excluded due to limitations in counting at very high or very low colony numbers. For example, the WT DT40 at dilution 1:1 (untreated) was too confluent for accurate counting, and no colonies were found in the two biological replicates of DT40 K563R at a 10 μM cisplatin for cell dilution of 1:100. Consequently, these points were excluded.<br>4) For the Xenopus egg extract experiment in Extended Data Fig 9c, the 30-minute timepoint of ΔD2+D2KREE is not shown due to poor digestion of this sample
Replication	All protein expression, purification, labelling, monoubiquitination assays and DNA binding essays, were performed at least three times, as indicated in the text. CryoEM data sets for dsDNA-D2-I and ss-dsDNA-D2-I were collected and processed two or three times, respectively, obtaining similar results. The largest data set for each complex was selected for obtaining the final reconstructions.<br>For single-molecule imaging, on each experimental day, we captured ~10-30 DNA molecules (one by one) that had a proper contour length (16.5 um for lambda DNA) and appropriate mechanical properties (persistence length, stretch modulus characteristic to dsDNA). Each experiment was replicated in at least 3 independent sessions. The resulting force-distance curves are practically identical, and are easily reproducible. The number of replicates is indicated for each experiment.
Randomization	Randomization is not relevant to the assays and biochemical experiments performed in this study. For experiments in Xenopus egg extracts,

Randomization	we subjected common extracts to the treatments indicated in the text. Cellular studies were conducted on cells from common pools that were treated with increasing amounts of DNA-damaging agent. For biochemical assays, the same protein stock was used for a given experiment. For calculation of the Fourier Shell Correlation using RELION, particles were automatically split into two random halves by the software. Replicates of all experiments show the results are reproducible and are not subjected to researcher's bias.
Blinding	Blinding is not relevant to the experiments presented in this study. Cryo-EM, biochemical data and cellular data were collected and processed identically under the same experimental conditions in an unbiased manner, and sample information did not lead to bias on any sample during the analysis.

Reporting for specific materials, systems and methods

We require information from authors about some types of materials, experimental systems and methods used in many studies. Here, indicate whether each material, system or method listed is relevant to your study. If you are not sure if a list item applies to your research, read the appropriate section before selecting a response.

Materials & experimental systems

n/a	Involved in the study
☐	☒ Antibodies
☐	☒ Eukaryotic cell lines
☒	☐ Palaeontology and archaeology
☐	☒ Animals and other organisms
☒	☐ Clinical data
☒	☐ Dual use research of concern
☒	☐ Plants

Methods

n/a	Involved in the study
☒	☐ ChIP-seq
☒	☐ Flow cytometry
☒	☐ MRI-based neuroimaging

Antibodies

Antibodies used	Histone H3 (ab1791, Abcam) used at 1:4000 dilution.<br>xlFANCD2 against N-terminal peptide (1-172aa) of xlFANCD2, described in https://doi.org/10.1016/ j.cell.2008.08.030. For western blot we used the serum as described in the reference (used in a 1:6000 dilution). For immundepletions we affinity purified the serum. This was a custom antibody raised at the Pocono Rabbit farm and Laboratory and a gift of the Walter lab (Harvard Medical School).
Validation	The Histone H3 antibody is a commercial antibody validated for Xenopus laevis Histone H3.<br>The xlFANCD2 antibody was previously validated in Xenopus egg extract (Knipscheer et al. Science 2009).

Eukaryotic cell lines

Policy information about cell lines and Sex and Gender in Research

Cell line source(s)	Sf9, Oxford Expression Technologies Ltd, Cat No. 600100.<br><br>DT40 is an avian leukosis virus (ALV) induced bursal lymphoma cell line derived from a Hyline SC chicken. Obtained from Dr John Young at the Institute of Animal Health, Compton, Berkshire UK. Also commercially available at https://www.atcc.org/ products/crl-2111
Authentication	No authentication of the Sf9 cell line was performed.<br>DT40 cell line was authenticated by Bu-1a and b expression and immunoglobulin gene sequences.
Mycoplasma contamination	All cell lines were tested for mycoplasma. No mycoplasma contamination was detected.
Commonly misidentified lines<br>(See ICLAC register)	No commonly misidentified cell lines were used in this study.

Animals and other research organisms

Policy information about studies involving animals; ARRIVE guidelines recommended for reporting animal research, and Sex and Gender in Research

Laboratory animals	Female Xenopus laevis frogs used in this study were older than 2 years and obtained from Nasco
Wild animals	The study did not involve wild animals.
Reporting on sex	Xenopus egg extracts were produced from the eggs of Xenopus laevis frogs and therefore this study only involved the use of female frogs.
Field-collected samples	This study did not involve field-collected samples.

Ethics oversight	All animal procedures and experiments were performed in accordance with national animal welfare laws and were reviewed by the Animal Ethics Committee of the Royal Netherlands Academy of Arts and Sciences (KNAW). All animal experiments were conducted under a project license granted by the Central Committee Animal Experimentation (CCD) of the Dutch government and approved by the Hubrecht Institute Animal Welfare Body (IvD), with project license number AVD80100202216633.

Note that full information on the approval of the study protocol must also be provided in the manuscript.
